# Paid Care Services and Transitioning Out of the Community among Diverse Older Adults with Dementia

**DOI:** 10.1093/geroni/igab046.3421

**Published:** 2021-12-17

**Authors:** Maria Roche-Dean, Sol Baik, Heehyul Moon, Norma Coe, Anna Oh, Laura Zahodne

**Affiliations:** 1 Western Michigan University, Battle Creek, Michigan, United States; 2 University of Maryland School of Social Work, Baltimore, Maryland, United States; 3 University of Louisville, university of Louisville, Kentucky, United States; 4 University of Pennsylvania, Philadelphia, Pennsylvania, United States; 5 San Francisco VA Health Care System, San Francisco, California, United States; 6 Clinical Science, Ann Arbor, Michigan, United States

## Abstract

Objectives: Paid care provided in the home or through community organizations includes important support services for older adults with dementia such as cleaning and personal care assistance. These services could delay the transition to long-term care, but access may differ across sociodemographic groups. This study examined the relationship between paid care and transitioning out of the community among diverse older adults with dementia. Methods: Using data from 303 participants (29.4% Black) with probable dementia in the National Health and Aging Trends Study (2011-2019), subdistribution hazard models estimated the association between receiving paid care at baseline and the probability of transitioning out of the community over the next eight years. Covariate selection was guided by the Andersen model of healthcare utilization. Results: Paid care was associated with lower risk of transitioning out of the community (SHR = 0.70, 95% CI [0.50, 0.98]). This effect was similar after controlling for predisposing factors and most prominent after controlling for enabling and need for services factors (SHR = 0.63, 95% CI [0.42, 0.94]) and was only evident among Whites. There were no racial differences in the use of paid care, but Black participants were less likely to transition out of the community than Whites despite evidencing greater care needs. Discussion: Paid care services may help delay transitions out of the community. Future research should seek to explain racial differences in access to and/or preferences for home-based, community-based, and residential care.

